# Optimizing Electrochemical Microprinting of Conducting Polymers: Scanning Electrochemical Cell Microscopy (SECCM) Coupled with Conveyor‐Belt Surface Analysis

**DOI:** 10.1002/smtd.202501781

**Published:** 2025-12-03

**Authors:** Noah Al‐Shamery, Dimitrios Valavanis, Bethanie Dean, Anna Dettlaff, Michał Sobaszek, Robert Bogdanowicz, Paul Wilson, Pooi See Lee, Patrick R. Unwin

**Affiliations:** ^1^ School of Materials Science and Engineering Nanyang Technological University 50 Nanyang Ave Singapore 639798 Singapore; ^2^ Department of Chemistry University of Warwick Coventry CV4 7AL UK; ^3^ Analytical Science CDT University of Warwick Coventry CV4 7AL UK; ^4^ Faculty of Chemistry Gdańsk University of Technology 11/12 Narutowicza Str. Gdańsk 80–233 Poland; ^5^ Faculty of Electronics Telecommunications and Informatics Gdańsk University of Technology 11/12 Narutowicza Str. Gdańsk 80–233 Poland

**Keywords:** carbon nanowalls, microfabrication, polypyrrole, scanning electrochemical cell microscopy (SECCM), sensor arrays

## Abstract

Scanning electrochemical cell microscopy (SECCM) is a versatile tool for localized electrochemical mapping, material modification, and microfabrication. In its hopping mode, the pipette‐based system confines reactions to the meniscus contact area, allowing precise deposition control. Here, an SECCM‐driven strategy for polypyrrole (PPy) microfabrication using phosphate buffer as the electrolyte, combined with an intermediate cleaning step to remove side products and prevent pipette clogging, is reported. This approach enables the production of uniform, circular PPy deposits with high reproducibility on gold substrates. A multi‐microscopy “conveyor‐belt” analysis – combining SEM, AFM, EDX, and Raman spectroscopy – reveals that phosphate ions intercalate into the PPy matrix during polymerization, as also seen in bulk studies. This intercalation is found to be reversible via post‐deposition rinsing. Furthermore, this work demonstrates that cyclic voltammetry‐based deposition enables patterned PPy growth on complex surfaces such as boron‐doped carbon nanowalls, overcoming surface charge and wetting challenges. These findings expand the applicability of SECCM for 2D conducting polymer micro‐/nanofabrication on both flat and structurally complex substrates.

## Graphical abstract

Scanning electrochemical cell microscopy (SECCM) enables precise and reproducible microprinting of polypyrrole. Using phosphate buffer and an intermediate cleaning step, it produces uniform, circular deposits, validated by a conveyor‐belt characterization workflow (SEM, AFM, EDX, Raman), revealing phosphate intercalation. Furthermore, it prints on rough boron‐doped carbon nanowalls using cyclic voltammetry, overcoming wetting/charge challenges and advancing the fabrication of micro/nanoscale sensor arrays.

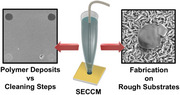

## 1 Introduction

Among scanning electrochemical probe microscopes, scanning electrochemical cell microscopy (SECCM) is distinct in the usage of a liquid meniscus formed at the tip of a pipette probe to limit electrochemical reactions to a confined and defined wetted area on the surface of a substrate.^[^ ^1^, ^2^, ^3^, ^4^ ^]^ Through this localized interaction, SECCM is not only used for mapping (photo)electrochemical surface properties and topographies, and for local surface manipulation,^[^ ^5^, ^6^ ^]^ but also for performing precise material deposition and modification.^[^ ^6^, ^8^, ^9^, ^10^, ^11^ ^]^ The availability of pipette probes with one,^[^ ^12^ ^]^ two,^[^ ^7^, ^13^ ^]^ or more,^[^ ^14^ ^]^ channels provides further possibilities for mixing and manipulating the solution composition, which can be particularly important for the local deposition of chemical species.^[^ ^7^ ^]^ SECCM, and meniscus‐based techniques more generally, have been used for metal electrodeposition,^[^ ^15^, ^16^, ^17^, ^18^, ^19^, ^20^ ^]^ the production of crystalline materials,^[^ ^21^ ^]^ chemical grafting of surfaces,^[^ ^22^, ^23^ ^]^ and patterning of surfaces with polymeric materials.^[^ ^24^, ^25^, ^26^, ^27^, ^28^ ^]^ SECCM has been applied to form deposits on mostly flat, metal or carbon substrates, and previous works have shown the technique to be effective in creating deposits and patterns of conducting polymers like polyaniline,^[^ ^24^ ^]^ poly(3,4‐ethylenedioxythiophene) (PEDOT),^[^ ^27^ ^]^ and polypyrrole (PPy).^[^ ^29^ ^]^

**Figure 1:**
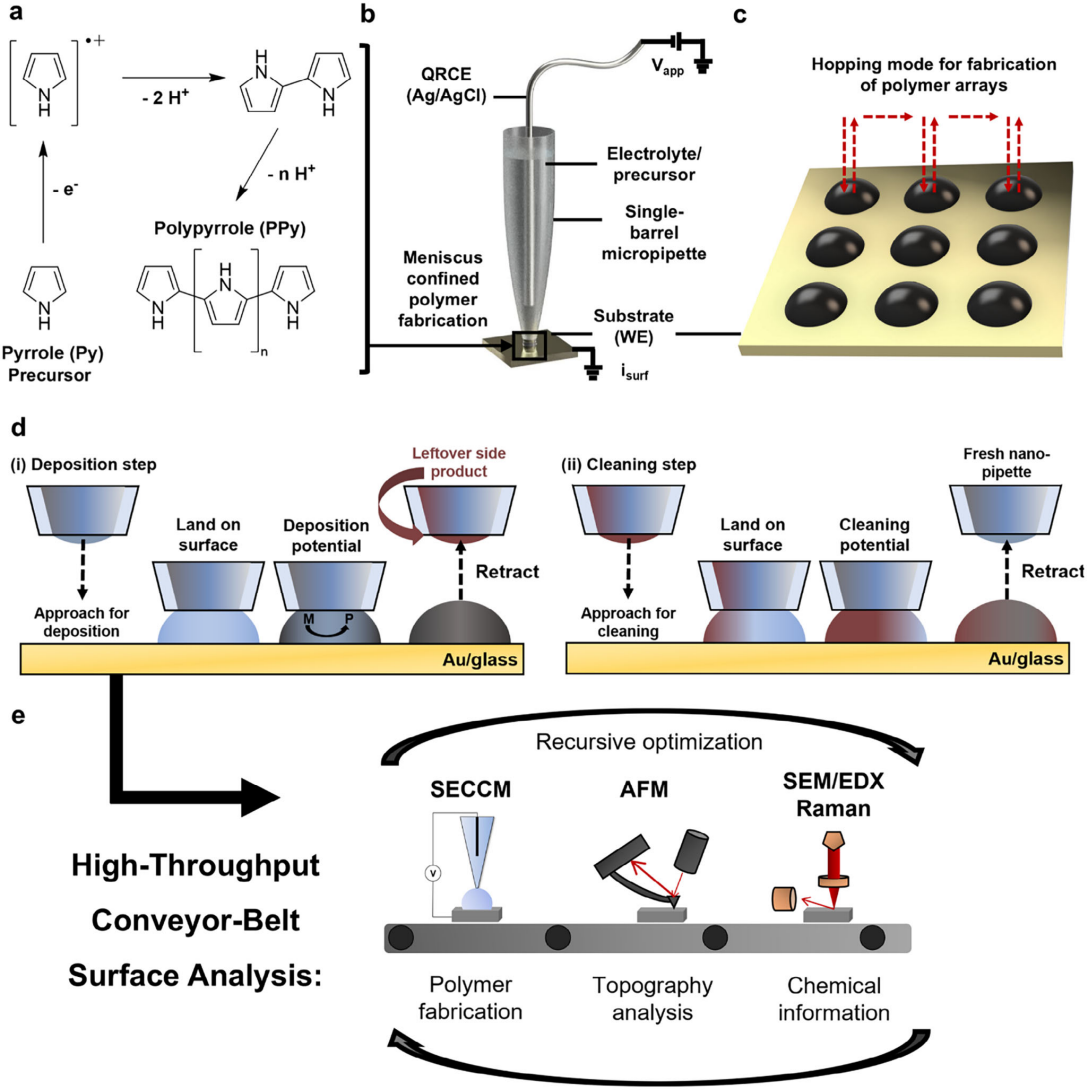
a) Reaction mechanism of pyrrole electropolymerization. Schematics of: b) the meniscus‐confined microfabrication of PPy using SECCM and c) the pipette trajectory during SECCM scan‐hopping for electrochemical polymer fabrication at every hop. d) SECCM protocol whereby the meniscus is brought into contact with the surface, a constant potential is applied to polymerize the monomeric species *M* to the polymeric species *P* for a deposition step (i); followed by the tip being withdrawn and proceeding to the next hop for a cleaning step (ii), where no polymerization occurs. e) Multi‐microscopy “conveyor‐belt” approach employed in this work, using different spectroscopy and microscopy techniques in iterations to optimize deposition conditions.

A schematic set‐up of the meniscus‐confined conducting polymer fabrication using single‐channel SECCM used in this work is given in **Figure** **1**, showing how polymer deposition is conducted. The fabrication of polymer is typically achieved using either cyclic voltammetry (CV) or by using chronoamperometry (*i*‐*t* deposits).^[^ ^24^ ^]^

This work focuses on the optimization of the fabrication and local analysis of PPy as a model system. As shown in Figure 1a next to the experimental set‐up (Figure 1b), the polymerization of pyrrole (Py) to PPy is an oxidative mechanism, involving the formation and dimerization of radical cations, and the further oxidation of these dimers/oligomers to produce polymer chains.^[^ ^30^ ^]^ The meniscus‐confined fabrication of polymer spot arrays (Figure 1c) is controllable via the overpotential and the duration of the applied potential.^[^ ^31^ ^]^ Despite SECCM allowing the nanofabrication of polymer arrays, control of the diameter and height of the deposits may be challenging.^[^ ^27^ ^]^ Additionally, rough and porous substrates, which may be of interest for preparing localized polymer composites on high surface area substrates, complicate the deposition process due to the more ill‐defined wetted area, and increased tip crashes caused by inhomogeneity of the surface.^[^ ^28^ ^]^ Thus, there is a clear need for improving the control and substrate selection for SECCM polymer deposition techniques.

In this study, we address the said challenges by first investigating the deposition of PPy on gold‐coated glass slides sputtered with an adhesion promoter followed by gold (hereby referred to as Au/glass) under different conditions, mainly by constant potential application and subsequent cleaning steps (Figure 1d), followed by iterative structural and chemical analysis. Using this information, we optimize the deposits by introducing regular cleaning steps, where leftover material is removed from the pipette, to mitigate the issues of pipette‐blockage through side‐product formation. Afterwards, we repeat the structural/chemical analysis and further tune the deposit and cleaning step conditions, until a satisfactory quality of fabricated nanomaterial is obtained. A schematic of this iterative multi‐microscopy “conveyor‐belt” approach, first described by Martín‐Yerga et al.^[^ ^32^ ^]^ can be seen in Figure 1e. Finally, we apply these strategies to enable deposits on a representative rough substrate (boron‐doped carbon nanowalls, B:CNW).^[^ ^33^ ^]^

## 2 Results and Discussion

### 2.1 Optimization of the Deposition Protocol

The following experiments used a *ca*. 2 µm diameter single‐barrel pipette tip, unless stated otherwise. First, without including any polymer precursor, the operating potential windows of two different aqueous electrolytes were determined: 0.05 M phosphate buffer (PB) and 0.05 M potassium nitrate (KNO_3_). These were selected as fairly common electrolytes in SECCM experiments. Figure S1 (Supporting Information) demonstrates that in the CV data for the aqueous systems, the oxygen evolution reaction (OER) and subsequent oxidative decomposition of the phosphate electrolyte occurred above a potential of *E*_OER_ = 1.0 V vs Ag/AgCl_QRCE_ (see Experimental Section).

Using the PB electrolyte and 10 mM Py monomer, *i*‐*t*‐deposits with an applied polymerization potential of 1.2 V upon surface contact for 200 ms, led to inconsistent diameter deposits due to increased wetting. Side product formation like pyrrole rings with carbonyl and hydroxyl groups introduced via overoxidation can also be problematic.^[^ ^34^ ^]^ This is seen in the SEM micrographs (coming from *i*‐*t*‐curve deposits as shown in Figure S2a, Supporting Information) depicted in Figure S2b (Supporting Information). The largest difference between deposit diameters was determined to be 8.0 µm, with the average diameter being 4.0 µm with a standard deviation of 1.4 µm. Based on this observation and the CV potential window experiments, subsequent *i*‐*t*‐deposits were performed at a polymerization potential of 1.0 V.^[^ ^35^ ^]^ This polymerization potential was chosen not only to remain below the onset of the oxygen evolution reaction observed in the control CVs, but also in line with bulk literature reports identifying 0.9–1.0 V (vs Ag/AgCl/KCl_sat._) as the upper stability limit for PPy growth on Au substrates.^[^ ^36^ ^]^ Applying 1.0 V (vs Ag/AgCl_QRCE_) for limited durations within the confined SECCM meniscus enables reproducible deposition while avoiding overoxidation and polymer degradation, as will be shown by post‐deposition spectroscopy analysis. SEM micrographs of polymer arrays prepared within the potential limits, using both cyclic voltammetry and amperometry, are given in Figure S3 (Supporting Information) with corresponding electrochemical data.

**Figure 2:**
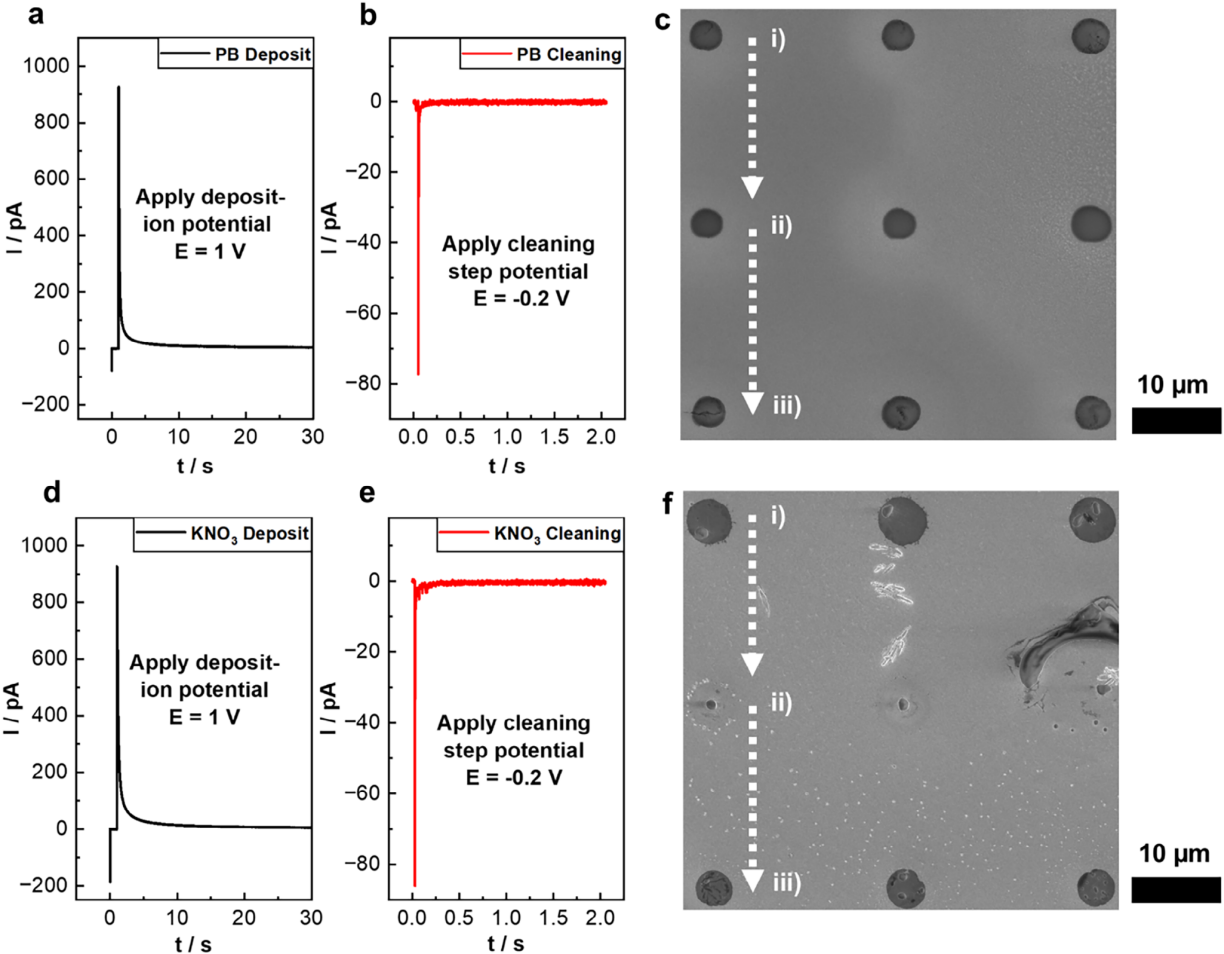
a) *i*‐*t*‐curves of a representative approach (*E* = ‐0.2 V versus Ag/AgCl_QRCE_) and deposit (*E* = 1.0 V vs Ag/AgCl_QRCE_) of a PPy deposition using an aqueous phosphate buffer solution electrolyte (*c* = 0.05 M). b) Representative cleaning step *i*‐*t*‐curve at a holding potential (*E* = ‐0.2 V vs Ag/AgCl_QRCE_) and c) the corresponding SEM micrograph of deposits i) and iii) and cleaning steps ii). d–f) show the same type of data for a PPy deposition, but using an aqueous potassium nitrate buffer solution electrolyte (*c* = 0.05 M) on Au. In (f), a clear difference between deposits i) and iii), and cleaning steps ii) is observable. All of the displayed measurements used a *ca*. 2 µm diameter tip, an approach rate of 0.5 µm s^−1^, and a retract rate of 3 µm s^−1^.

To further improve the consistency in‐between deposits, a cleaning step was introduced. After each *i*‐*t*‐deposit, the following spot was maintained at the approach potential of −0.2 V, which was applied for varying times depending on the step increment time, instead of immediately switching to the polymerization potential. No reaction occurred at this potential, so that any side‐products from the previous deposit, as well as potential tip blockage, tended to be removed. Then, the pipette was moved to the next spot where a fresh pipette approach and application of the polymerization potential (1.0 V) was implemented. **Figure** **2**a,b shows *i*‐*t*‐curves of a set of approaches and polymer deposits using the PB electrolyte, followed by a cleaning step, for a 6 × 6 array containing 18 PPy deposition sites and 18 cleaning steps, arranged sequentially with a hopping distance of 20 µm between positions. Subtracting the baseline current before the potential application from the current value after the deposit (a) and cleaning step (b) respectively, gives a leftover current that can be used as an indicator to determine if a Faradaic reaction has occurred or not. This gives a leftover current of ≈ 3–5 pA for (a) and currents < 0.1 pA for (b), indicating that no detectable reaction occurred during the cleaning step.

To maintain high throughput and minimize drift, the cleaning step was carried out one hop distance after each deposit. Our approach presented here preserves tip stability and reproducibility between consecutive deposition steps.

The camera image in Figure S2 (Supporting Information), displaying a close‐up of the microscale pipettes used in this work, shows that the polymer arrays are visible in operando. A 3×3 SEM micrograph of 9 deposits from the discussed cleaning‐step PPy array is depicted in Figure 2c, with each column representing a sequence (from top to bottom) of: i) deposit, ii) cleaning step, iii) deposit. The complete 6×6 array SEM micrograph is shown in Figure S4a (Supporting Information). For the PB electrolyte deposits of PPy, no morphological difference is observed between deposits and cleaning steps in the SEM micrograph. The PB‐electrolyte PPy deposits obtained from the procedure with cleaning steps had an average diameter of 3.6 ± 0.7 µm, with a maximum deviation between the largest and smallest deposit of 3.2 µm. This is significantly smaller and improved compared to the previously discussed procedure without cleaning steps.

To show the electrolyte influence on the deposits and cleaning steps, the experiment was repeated with the 0.05 M KNO_3_ electrolyte, an electrolyte also commonly found in the literature for SECCM mapping and fabrication experiments.^[^ ^16^, ^37^ ^]^ Figure 2d–f from left to right shows the (d) deposit and (e) cleaning step *i*‐*t*‐curves, and (f) a 3×3 SEM micrograph excerpt of the complete 6×6 array (Figure S4b, Supporting Information). For additional clarity, the corresponding *i*‐*t* curves were plotted exemplarily alongside the *z*‐position and applied electrode potential as well, which is depicted in Figure S5 (Supporting Information) ((a) for the deposit and (b) for the cleaning step) for the KNO_3_ sample. The deposits resulting from the procedure with cleaning steps had an average diameter of 4.3 ± 0.9 µm. For the experiment using KNO_3_‐based electrolyte, clear differences between the morphology of the deposits and cleaning steps can be observed. The deposits show the expected dark contrast in the area wetted by the meniscus, with small crystalline protrusions apparent within them. This behavior is not observed for the PB‐electrolyte deposits in Figure 2c. Despite this, the *i*‐*t*‐curves of the deposit and cleaning step have clear similarities between the two electrolytes, such as similar maximum/minimum currents and decay time. The cleaning steps do not have dark contrast, but do show a ring of crystallized material resulting from the evaporation of the KNO_3_ electrolyte droplet. Small crystal protrusions are visible in the centers of the cleaning steps with an average size of 1.2 ± 0.4 µm, indicating the potential presence of crystallized electrolyte.

### 2.2 Understanding the Chemistry and Mechanism of the Deposits

**Figure 3:**
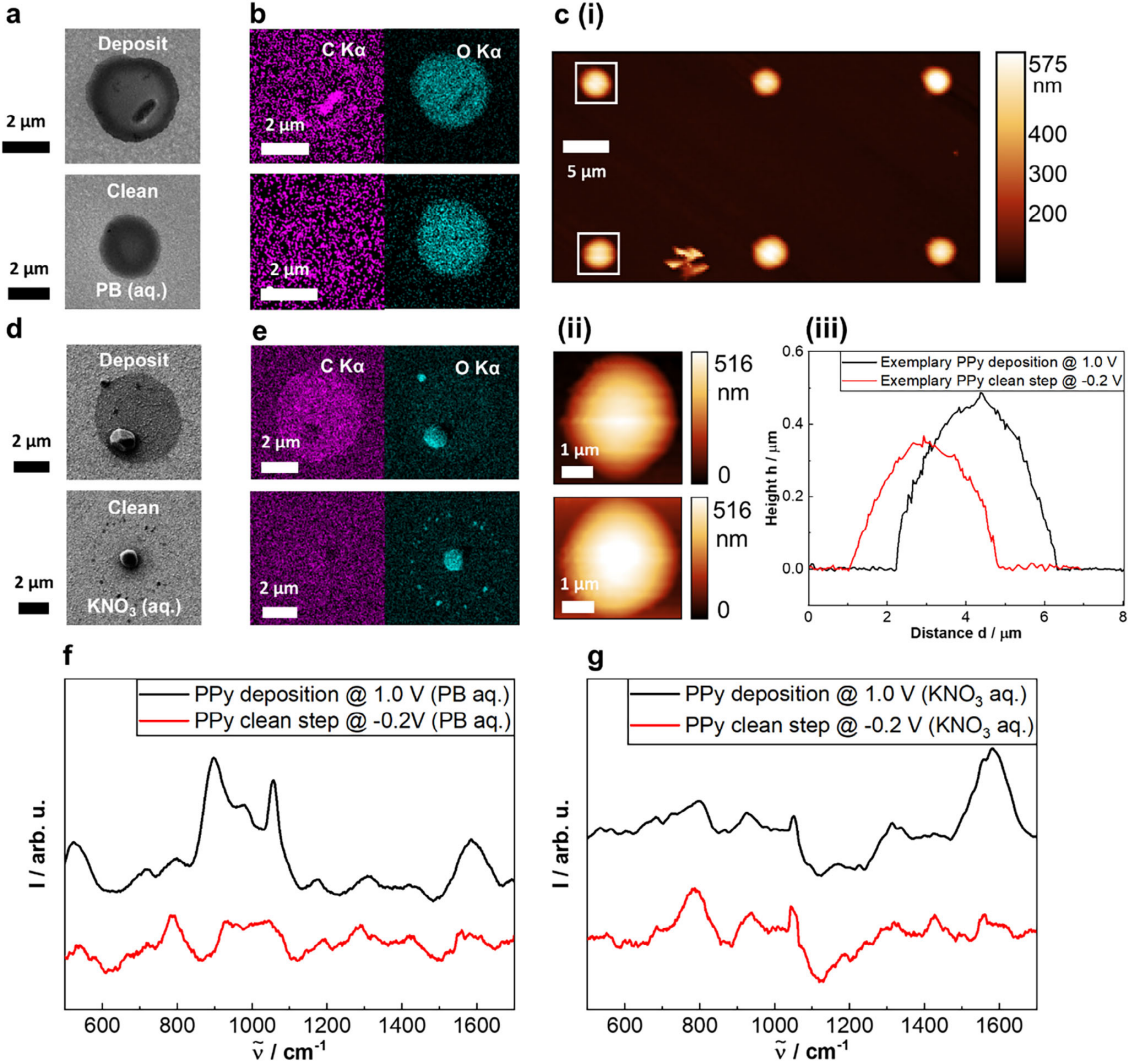
a) SEM micrographs of a PPy deposit (top) and cleaning step residue (bottom) on Au using PB electrolyte with (b) corresponding EDX images of C and O counts. The C‐ and O‐EDX images employ the same scale bars for the same respective samples (2 µm). c) (i) shows three PPy depositions in PB electrolyte (top row) and cleaning steps (bottom row), while (ii) displays close‐ups of the boxed deposit (top) and cleaning step (bottom) with corresponding height profiles seen in (iii). d) SEM micrographs of a PPy deposit (top) and cleaning step (bottom) on Au using KNO_3_ electrolyte with (e) corresponding EDX images of C and O counts. f) Raman spectra of representative spots of the samples using PB electrolyte and (g) KNO_3_ electrolyte are displayed. At least 3 different spots were analyzed for each sample‐type. Minor differences in the background brightness between the EDX maps for the phosphate and nitrate electrolyte samples ((b) and (e)) originate from different acquisition sessions. These variations do not affect the interpretation of the elemental distributions.

Adapting the multi‐microscopy “conveyor‐belt” approach, deposits were characterized by a series of microscopy and spectroscopy techniques. **Figure** **3**a shows SEM micrographs for a PB‐electrolyte PPy deposit (top) and cleaning step (bottom) from the sample in Figure S4a (Supporting Information). The SEM data show that the deposit from the cleaning step appears flatter than the PPy deposit, and does not contain any surface protrusions. The EDX analyses of carbon and oxygen as shown in Figure 3b reveals that for PB electrolyte PPy deposits, the protrusions are local agglomerations of carbon‐rich material, as the C signal depicts high intensity whilst the O signal has low intensity at the location of the protrusion. Looking at the chemical structure of PPy as displayed in Figure 1a, the conjugated C═C bond backbone is expected to cause a high intensity carbon signal. Interestingly, the C signal only shows high intensity on this protrusion and on the circumference of the deposit, whereas the O distribution can be found throughout the deposit. Similarly, the O‐signal intensity is strong throughout the cleaning step deposit. This could potentially indicate the presence of leftover electrolyte salt in the PPy polymer matrix.

Through colocalized AFM analysis, height profiles and average dimensions of PPy deposits and cleaning steps were obtained. Three deposits (top row) and cleaning steps (bottom row) are shown in Figure 3c (i), with corresponding AFM close‐ups in (ii) and height profiles in (iii). Measurements extracted using Gwyddion^[^ ^38^ ^]^ gave average diameters and heights of $\bar{d}$_deposit_ = (3.7 ± 0.2) µm and $\bar{h}$_deposit_ = (0.6 ± 0.1) µm. For cleaning steps, values were $\bar{d}$_clean_ = (3.6 ± 0.2) µm, and $\bar{h}$_clean_ = (0.4 ± 0.1) µm. While diameters were similar, consistent with meniscus confinement, cleaning steps were consistently lower in height, likely due to shorter potential application times (30 s vs 1 s), in agreement with SEM observations.

Figure S6a (Supporting Information) shows height profiles from the central column in Figure 3c (i): a cleaning step, a deposit, and a subsequent cleaning step. The slightly reduced heights before and after deposition (*h*_1_ = 0.42 µm and *h*_2_ = 0.41 µm) suggest minimal material transfer. AFM of KNO_3_‐based PPy (Figure S6b, Supporting Information) revealed protrusions with $\bar{h}$_clean, max_ = (0.5 ± 0.2) µm, with larger height contrasts than in PB, suggesting more random material distribution for PB.

Further insight into these protrusions comes from Figure 3d,e. SEM morphology and EDX oxygen signals suggest that both deposits and cleaning steps in KNO_3_ electrolyte contain crystallized salt. Notably, cleaning steps show electrolyte residue at the meniscus edge, likely due to unhindered salt creep during droplet drying. Finally, strong carbon signals in deposits, but not in cleaning step residues, confirm that polymerization (at 1.0 V) is essential for forming carbon‐rich PPy material.

Raman spectra for PB‐electrolyte PPy deposits and cleaning steps can be seen in Figure 3f, and for KNO_3_‐electrolyte deposits and cleaning steps in Figure 3g. The black traces represent the full Raman spectrum for the PPy deposit, and the red traces are for the cleaning steps. It is clear that for the cleaning steps in PB electrolyte, the spectrum shows a broad arrangement of bands in the range of 200–1000 cm^−1^, corresponding to the presence of O─P and O─H vibrational bands,^[^ ^39^ ^]^ with an additional strong band ≈ 1050 cm^−1^ present in the KNO_3_‐electrolyte sample, corresponding to NO_2_ stretching modes.^[^ ^40^ ^]^ For both cleaning steps, no strong bands ≈ 1500 cm^−1^ are observed, confirming that polymeric material is not deposited.^[^ ^41^ ^]^ The characteristic pair of bands at 1600 and 1400 cm^−1^ correspond to conjugated C═C bonds, as found in the polymer backbone of polypyrrole. Strong bands in this region are only observed in the Raman spectra of the PPy deposits for the PB‐ and KNO_3_‐electrolyte samples.

For the deposits using PB electrolyte, peaks around 500 cm^−1^, 900 cm^−1^, and 1000 cm^−1^ are also observed in addition to the C═C double band pair. These bands are found in the Raman spectra of solutions containing phosphate ions, as discussed in the literature.^[^ ^42^ ^]^ This observation further confirms the hypothesis that phosphate ions have been integrated into the PPy matrix during deposition, aided by polymer swelling.^[^ ^43^, ^44^ ^]^

The adhesion and electrochemical stability of PPy layers are strongly dependent on both the applied potential range and the nature of the substrate. Previous studies have shown that bulk PPy films grown on gold remain stable during potential cycling up to ≈0.9–1.0 V versus Ag/AgCl/KCl_sat._, but tend to delaminate or “repulse” from the gold surface when the upper potential exceeds 1.0 V versus Ag/AgCl/KCl_sat._ due to overoxidation and the formation of surface oxides on gold.^[^ ^36^ ^]^ In our SECCM configuration, the limited deposition pulses (in the case of constant potential application) and fast scan rates (in the case of CV) applied in the confined meniscus contact minimize such degradation, preserving adhesion while achieving controlled polymer growth. Importantly, within the measured spectral range, no additional Raman bands were observed in the regions characteristic of oxidative or structural degradation, namely 1300–1400 cm^−1^ (C‐N^+•^ related species) and 1650–1700 cm^−1^ (carbonyl C═O stretching) after the deposit. The absence of new features in these regions confirms the applied deposition potential did not induce overoxidation or disruption of the conjugated PPy backbone, and that the polymer structure remained chemically intact under the employed conditions.^[^ ^41^ ^]^ Moreover, the use of phosphate buffer appears to strengthen interfacial stability, potentially through reversible ion intercalation and hydrogen‐bonding with the polymer backbone, as evidenced by the oxygen‐rich Raman and EDX signatures. This contrasts with deposits formed in nitrate electrolyte, where crystalline protrusions and poorer adhesion are observed. The micrometer scale of the experiment using PB seems to maintain PPy adhesion even at potentials near the polymerization threshold.

Through iterative modification of the deposit parameters in a narrow experimental space, motivated by spectroscopic findings, the quality of polymer deposits has been improved. Further possible deposit modifications by using nanoscale tips, different monomers, and rinsing the deposits with deionized (DI) water, are discussed in Note S5 (Supporting Information) with corresponding Figures S7–S11 (Supporting Information). Briefly, it was observed that cleaning steps could also be employed with a ∼200 nm pipette, and the beneficial effect of phosphate was found to not be unique to PPy, as shown by comparable SEM/EDX data for PB‐electrolyte polyaniline deposits. Additionally, rinsing with DI water effectively removes intercalated phosphate species from the deposits, as confirmed by Raman spectroscopy and SEM analysis.

### 2.3 SECCM‐Polymer Fabrication on Rough Surfaces

**Figure 4:**
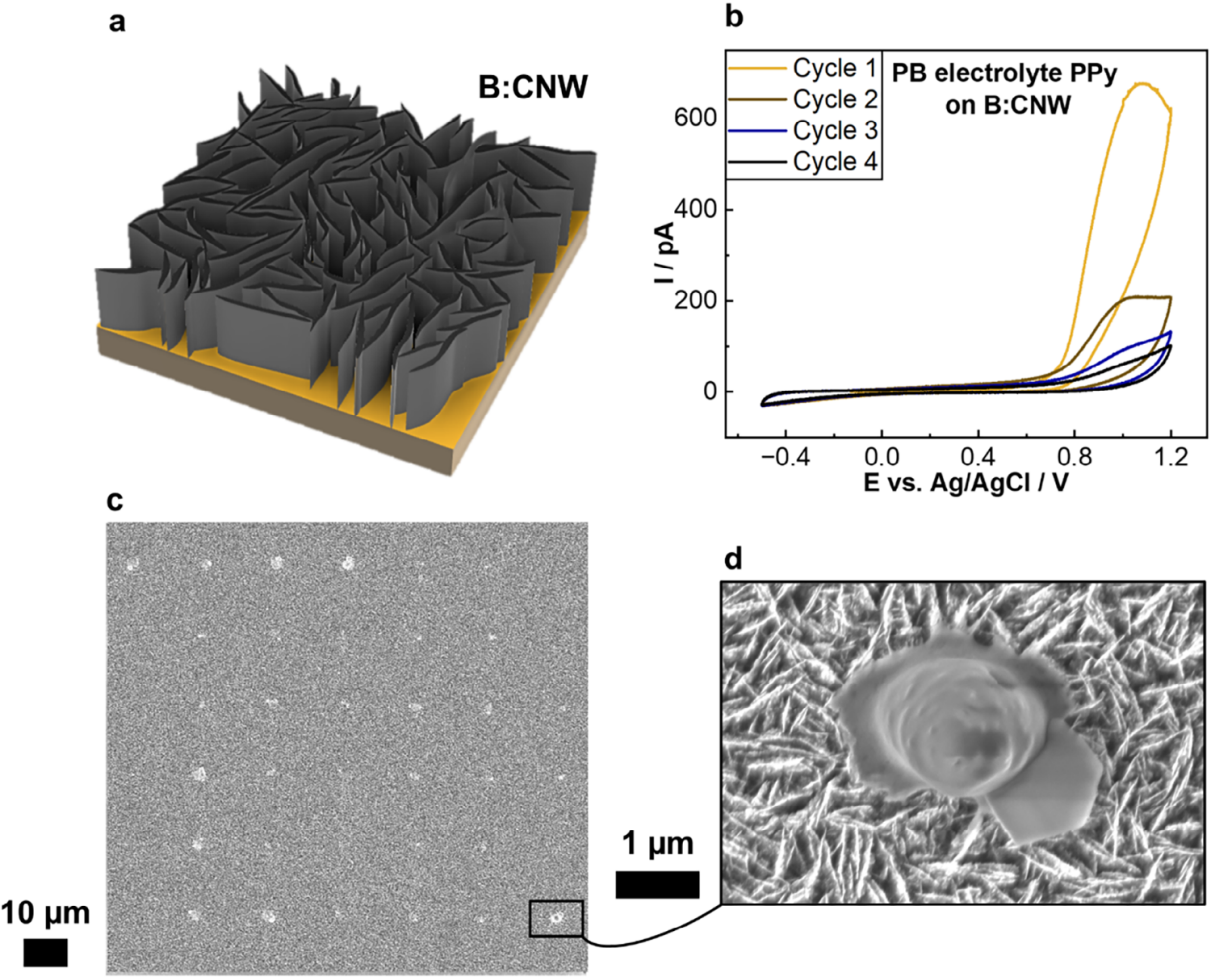
a) Schematic of the boron‐doped carbon nanowall‐structure. b) CV data of four cycle layer‐by‐layer deposition of PPy onto the B:CNW substrate at a scan rate of 1.0 V s^−1^ (*E*_start_ = −0.2 V; *E*_1_ = 1.2 V; *E*_2_ = −0.5 V vs Ag/AgCl_QRCE_). c) 6 × 6 array of approximately 2‐µm sized deposits of PPy on B:CNW (obtained from cyclic voltammetry deposits employing PB electrolyte). d) Close‐up of a single spot deposit from the array.

Finally, the pipette‐based microfabrication of PPy using SECCM was investigated using rough, maze‐like boron‐doped carbon nanowall (B:CNW) electrodes as substrates (**Figure** **4**a).^[^ ^45^ ^]^ Carbon nanowalls (CNW) are 2D, vertically self‐standing graphene multilayers. CNW films are usually grown using chemical vapour deposition methods onto substrates. Boron‐doped carbon nanowalls show enhanced electrical conductivity and specific sharp‐edged and at the same time flat structure.^[^ ^33^, ^46^ ^]^ Consequently, these structures can be effectively utilized for many electrochemical applications, including electrochemical sensors and energy storage devices, making B:CNW an interesting substrate for studying pipette‐based microfabrication on rough substrates.^[^ ^45^, ^47^, ^48^, ^49^ ^]^

To highlight the benefits and limitations of this substrate, contact angle measurements were performed, with the results presented in detail in Note S6 and Figure S12 (Supporting Information). B:CNW was shown to exhibit significantly greater hydrophobicity than Au across all tested analytes (134.6° ± 1.6° contact angle using DI) due to high surface charge stemming from the boron‐doping, making it well‐suited for precise meniscus‐confined polymer fabrication using SECCM.

First, bare PB electrolyte micropipette‐CV tests were performed on the B:CNW substrate, in order to elucidate the potential window that could be explored for this substrate type (−0.5 to 1.2 V vs Ag/AgCl_QRCE_). The results can be seen in Figure S13 (Supporting Information) with further discussion in Note S6 (Supporting Information). The *i*‐*t*‐deposit attempts with 200 ms application time of 1.0 V were unsuccessful, with some wetting at the landing sites observable but no polymer formation (Figure S14, Supporting Information, representative electrochemical data in (a) and SEM micrograph with close‐up inset in (b)). In contrast, the CV‐layer‐by‐layer deposition of PPy on B:CNW was performed successfully.

The CV‐graphs in Figure 4b shows the cyclic voltammetry performed at each spot of a 6×6 array of PPy deposits. After four cycles, the maximum current decreased from *i*_max_ = 680 pA to *i*_max_ = 100 pA, indicating the presence of a natural passivating layer during the deposition, previously observed in the literature for bulk PPy electropolymerization.^[^ ^35^ ^]^ The SEM micrograph in (c) and the close‐up of the boxed deposit in (d) revealed that PPy was successfully deposited on B:CNW with high reproducibility. This is the first report of polymer deposition on a rough substrate like B:CNW using SECCM. An average deposit diameter of $\bar{d}$_deposit_ = (2.0 ± 0.4) µm was observed from the SEM data. The EDX single‐spot analysis shown in Figure S15 (Supporting Information) confirms a similar behavior to the unrinsed PB‐PPy samples on Au/glass discussed in this work. The C Kα‐EDX image clearly displays the carbon‐rich nature of the B:CNW substrate. Cross‐section analysis and in situ tracking of the polymerization could provide further information about the polymerization mechanism in future work.

The combination of PPy with carbon‐based substrates is widely recognized for enabling efficient charge storage due to the coexistence of electric‐double‐layer and pseudocapacitive mechanisms.^[^ ^50^ ^]^ Recent studies have shown that PPy films conformally deposited on porous or fibrous carbon supports can deliver high areal and volumetric capacitance while maintaining excellent cycling stability. For example, PPy coatings grown by oxidative chemical vapor deposition on thermally activated carbon cloth exhibited capacitances approaching 300 mF cm^−2^ and stable performance over thousands of charge‐discharge cycles.^[^ ^50^ ^]^ The improved capacitive response in such hybrid electrodes arises from the synergistic combination of the high surface area and conductivity of carbon with the reversible redox activity of PPy. In this context, our PPy deposits on boron‐doped carbon nanowalls share many of the same interfacial advantages: enhanced adhesion, high electroactive surface exposure, and accessible ion‐transport channels. The spatially confined deposition we employ provides a route to locally tailor pseudocapacitive behavior at the microscale, which could be exploited in on‐chip micro‐supercapacitor designs or integrated electrochemical energy‐storage systems.^[^ ^51^ ^]^

Besides the potential applications, the combined electrochemical and morphological results show that the local deposition environment strongly affects deposit uniformity and composition. Raman and SEM/EDX analyses suggest that electrolyte composition influences the polymer doping level and oxidation state. The confined meniscus geometry and local surface charge further contribute to differences in morphology and adhesion. On B:CNW, polymer growth was achieved using cyclic voltammetry, suggesting that a dynamic potential profile better accommodates the interfacial conditions of this rough, conductive substrate.

## 3 Conclusion

This work establishes a practical and versatile approach for the precise microfabrication of conducting polymers on both flat and complex surfaces using SECCM. By integrating a simple yet effective cleaning step into the SECCM workflow, we achieved reproducible PPy arrays with regular 3–4 µm and 600 nm diameter deposits using 0.05 M phosphate buffer electrolyte. In contrast, deposition in 0.05 M KNO_3_ resulted in irregular growths and crystalline protrusions, confirming the critical role of electrolyte composition. The multi‐microscopy “conveyor‐belt” characterization (SEM/EDX, AFM, Raman) revealed that phosphate ions intercalate into the PPy matrix during meniscus‐confined polymerization, an effect previously only observed in bulk studies, and that this intercalation can be reversed post‐deposition by DI water rinsing. Most notably, we demonstrate that PPy can be patterned on rough, high‐surface‐charge substrates such as B:CNWs via cyclic voltammetry in a controlled, layer‐by‐layer manner. This overcomes long‐standing challenges related to porosity and wetting, and positions SECCM as a powerful platform for fabricating micro‐/nanoscale sensor arrays on unconventional surfaces.

## 4 Experimental Section

### Reagents and Materials

All electrolyte and monomer materials were purchased from Sigma–Aldrich, UK, and used as received. A detailed list of the reagents and materials used and how they were incorporated into the electrochemical set‐up using pipette probes, is given in Note S1 (Supporting Information). The experimental set‐up used for obtaining boron‐doped carbon nanowalls (B:CNW) substrates has been described in detail in the literature.^[^ ^52^, ^53^ ^]^ Further details of the fabrication are discussed in Note S2 (Supporting Information). Chloridized silver wires (diameter 0.125 mm, Goodfellow, UK) were employed as quasi‐reference counter electrodes (QRCEs). The potentials reported in this work are defined with reference to this Ag/AgCl_QRCE_, which behaves analogously to a conventional Ag/AgCl reference electrode in aqueous media, and has been shown to maintain excellent stability on the several hours time scale, under typical SECCM conditions.^[^ ^54^ ^]^ The parameters for the fabrication of each type of pipette have been discussed previously.^[^ ^55^ ^]^

### SECCM Instrumentation

All electrochemical experiments were performed using a home‐built SECCM instrument, as reported in previous works (Figure 1b).^[^ ^2^, ^4^, ^11^, ^56^ ^]^ The electrolyte‐filled single‐barrel pipette probe was mounted on a *z*‐piezoelectric positioner (P‐753.5 actuator and E‐665 controller, Physik Instrumente, Germany), and the Au working electrode (WE) was mounted on a *xy*‐piezoelectric positioner (Nano‐Bio300 stage and Nano‐Drive controller, Mad City Labs, USA). As seen in Figure 1b, all CV and chronoamperometry (*i*‐*t*) experiments were performed in the confined area of the meniscus cell between the WE surface and SECCM probe tip. Surface mapping and polymer array fabrication was performed using a standard *hopping mode* protocol.^[^ ^57^, ^58^ ^]^ Briefly, the SECCM single‐barrel probe approached the WE at a grid of predefined locations, with an independent electrochemical measurement being made at each spot. Through the modification of potential application time and potential value (window) parameters, arrays of polymers could be produced with different printing conditions at every spot.

Here, Au/glass and B:CNW substrates were analyzed by performing meniscus‐confined cyclic voltammetry on them. PPy arrays in varying grid sizes were fabricated using electropolymerization by CV (4 cycles both on Au/glass and on B:CNW, using the potential range obtained from analysis without monomeric material) and by chronoamperometry (with differing polymerization times from a few milliseconds to several seconds, and differing polymerization potentials around the oxidative polymerization potential of Py (1.0 V vs Ag/AgCl_QRCE_) depending on the studied properties of the deposition procedure). For measurements employing the *cleaning step* procedure, an additional 200 ms to 1 s deposition at −0.2 V versus Ag/AgCl_QRCE_ was performed in‐between polymer deposits, to remove leftover polymerization side products and reduce pipette blockage between sequential steps. The cleaning potential was selected so that no reaction occurred for the observed system.

Electrochemical data were processed using the MATLAB (release R2019b, The MathWorks, Inc., USA) software package. Data plotting was carried out using OriginPro (release 2023b, OriginLab, USA), and analysis of microscopy data was realized using ImageJ^[^ ^59^ ^]^ (release 1.54, open source) and Gwyddion^[^ ^38^ ^]^ (release 2.66, open source) software packages. Further specifications of the SECCM set‐up^[^ ^60^ ^]^ can be found in Note S3 (Supporting Information).

### Microscopic and Spectroscopic Characterization

Scanning electron microscopy (SEM) coupled with energy‐dispersive X‐ray spectroscopy (EDX), atomic force microscopy (AFM), and room temperature Raman spectroscopy were conducted on the substrates after polymer deposition. Contact angle measurements were performed to characterize the wetting of the employed electrolytes on the Au/glass and B:CNW substrates. Instrumentation details are presented in Note S4 (Supporting Information).

## Conflict of Interest

The authors declare no conflict of interest.

## Author Contributions

All authors listed have made an intellectual, substantial, and direct contribution to the work and approved it for publication. N.A.‐S., under the main supervision of P.U. and P.S.L., conceived the original work together with D.V. and P.U. D.V. advised on sample preparation and experimental planning, and assisted with SECCM and SEM/EDX experiments mainly performed by N.A.‐S. B.D., under the main supervision of P.W., further assisted with sample preparation, SEM/EDX, and AFM experiments. A.D. advised on the preparation and characterization experiments of the B:CNW samples and created the material schematic. M.S. directly prepared B:CNW samples under the supervision of R.B. N.A.‐S. performed the remaining material fabrication, characterization, and electrochemical testing. N.A.‐S. wrote the original draft of the article and Supporting information; all authors edited, reviewed, and agreed with the manuscript.

## Supporting information



Supporting Information

## Data Availability

The data that support the findings of this study are available from the corresponding author upon reasonable request.
