# Supplementary material for: Optimizing Electrochemical Microprinting of Conducting Polymers: Scanning Electrochemical Cell Microscopy (SECCM) Coupled with Conveyor‐Belt Surface Analysis
Source: Small Methods. 2025 Dec 3;10(1):e01781. doi: 10.1002/smtd.202501781 (PMC12790364; doi:10.1002/smtd.202501781)
Supplement: Supplementary file 1 — Supporting Information [file SMTD-10-e01781-s001.pdf]

Supporting Information

Optimizing Electrochemical Microprinting of Conducting Polymers: Scanning Electrochemical Cell Microscopy (SECCM) Coupled with Conveyor-Belt Surface Analysis

*Noah Al-Shamery[a,b], Dimitrios Valavanis[b], Bethanie Dean[b,c], Anna Dettlaff[d], Michał Sobaszek[e], Robert Bogdanowicz[e], Paul Wilson[b], Pooi See Lee[a], and Patrick R. Unwin[b]**

[a]School of Materials Science and Engineering, Nanyang Technological University, Singapore, 50 Nanyang Ave, 639798 Singapore

[b]Department of Chemistry, University of Warwick, CV4 7AL Coventry, United Kingdom

[c]Analytical Science CDT, University of Warwick, CV4 7AL Coventry, United Kingdom

[d]Gdańsk University of Technology, Faculty of Chemistry, 11/12 Narutowicza Str., 80-233, Gdańsk, Poland

[e]Gdańsk University of Technology, Faculty of Electronics, Telecommunications and Informatics, 11/12 Narutowicza Str., 80-233, Gdańsk, Poland

*Corresponding author
Patrick R. Unwin: p.r.unwin@warwick.ac.uk

Contents list: This PDF file includes: Supporting Notes 1-6; Supporting Figures S1 to S14.

Supporting Note 1: Detailed list of used reagents and materials

Supporting Note 2: Preparation of B:CNW substrates

Supporting Note 3: Additional specifications on the SECCM set-up

Supporting Note 4: Details of the spectroscopy/microscopy instrumentation

Supporting Note 5: Further tuning the deposits: Nanoscale pipettes, PANI, DI-rinsing

Supporting Note 6: Details regarding the additional characterization of B:CNW

Figure S1: Electrolyte CV data

Figure S2: Data for high potential deposits and additional camera images

Figure S3: SEM micrographs of fabricated polymer arrays

Figure S4: Complete SEM micrographs of PPy arrays using different electrolytes and cleaning steps

Figure S5: Deposit and cleaning currents alongside *z*-positions and potential waveforms

Figure S6: Additional height profile and single spot AFM data

Figure S7: SECCM *i*-*t*-curve of nanoscale PPy deposit and cleaning step

Figure S8: Nanoscale and DI-rinsed PPy

Figure S9: EDX characterization of nanoscale PPy

Figure S10: EDX characterization of PANI deposit

Figure S11: Additional characterization of rinsed PB-electrolyte PPy deposits

Figure S12: SEM and contact angle tests of B:CNW (and Au) substrates

Figure S13: CV potential window checking of B:CNW

Figure S14: Electrochemical and SEM data of *i*-*t* deposits of PPy on B:CNW

Figure S15: EDX single-spot analysis of PB-electrolyte PPy deposit on B:CNW

Supporting Note 1: Detailed list of used reagents and materials

Potassium phosphate monobasic (KH_2PO_4, ≥99%) and potassium phosphate dibasic (K_2HPO_4, ≥98%) were dissolved in ultrapure deionized water (DI, PURELAB Chorus, ELGA, UK) to prepare a 0.05 M phosphate buffer (PB) solution, adjusted to pH = 7.4 using potassium hydroxide (KOH, ≥85%). Potassium nitrate (KNO_3, ≥99%) was dissolved in DI to prepare 0.05 M electrolyte solutions. Each electrolyte was mixed with pyrrole (Py, C_4H_5N, 98%) or aniline hydrochloride (C_6H_8ClN, 99%) to prepare 10 mM precursor solutions for electropolymerization. Circular, 0.16 mm - 0.19 mm thick glass coverslips (Academy, UK) were rinsed and dried before sputtering (nanoPVD-S10A, Moorfields Nanotechnology, UK) with 50 nm of Ti as an adhesion promoter, and 200 nm of Au, to prepare Au/glass substrates. Both Au/glass and B:CNW substrates were secured on a circular stainless steel SEM sample holder using an Au pin, and silver conductive paint (RS components, UK) to further improve connection. They acted as the working electrode (WE) for SECCM. Two different sizes (end aperture) of pipettes were prepared (*ca.* 200 nm and *ca.* 2 μm diameter) from borosilicate filamented glass capillaries (BF120-69-10, World Precision Instruments Inc., USA) using a laser pipette puller (P-2000, Sutter Instrument, USA). The 200 nm diameter pipettes were used for nanoscale PPy-fabrication using a PB-precursor electrolyte, whereas the *ca.* 2 μm diameter pipettes were used for all other experiments. Before inserting the QRCE into the pipette, the pipette was filled with the electrolyte/precursor solution using a MicroFil syringe (World Precision Instruments Inc., USA).

Supporting Note 2: Preparation of B:CNW substrates

B-doped carbon nanowall electrodes were deposited onto p-type (100) silicon substrates using a Microwave Plasma-Assisted Chemical Vapor Deposition (MPCVD) system (AX5200S, Seki, Japan). The 10×10 mm silicon plates were cleaned sequentially by ultrasonic bath in acetone and 2-propanol for five minutes each. Subsequently, the substrates were sonicated for 25

minutes in a water-based nanodiamond slurry (4–5 nm nanodiamond particles). During the deposition process, the gas mixture consisted of 8% methane, 2000 ppm B/C from diborane, with hydrogen as the balance, reaching a total flow rate of 300 sccm. Nitrogen was introduced as a growth promoter for BCNW formation. The deposition was carried out with a substrate temperature of 700 °C, a process pressure of 50 Torr (6.66 kPa), and a growth duration of 5 hours, giving walls with an approximate height of 80 nm and a width of approximately 200 nm.

Supporting Note 3: Additional specifications on the SECCM set-up

By using an analyte that did not require a precursor for electrochemical activity, surface mapping could be performed by monitoring the current response either under a constant potential or during a cyclic voltammetry scan. At each measurement point, the SECCM probe approached the working electrode (WE) surface until the liquid meniscus formed at the pipette tip made contact. This contact was detected electrochemically when the surface current exceeded a user-defined threshold, set above the system's noise level, at a chosen approach potential. Upon detection, the probe's vertical movement (controlled by the *z*-piezo positioner) halted, and the final *z*-position was recorded. Repeating this process across an array of points allowed simultaneous construction of an electrochemical activity map and a corresponding topographical map of the WE surface based on the *z*-piezo displacement.

The SECCM system was placed on a vibration isolation platform (Minus K, USA) positioned inside an aluminum Faraday cage, and lined with thermal isolation panels and acoustic foam. The QRCE potential control (with respect to ground) and the current flowing at the WE (held at a common ground), were determined by employing a home-built electrometer head. The current was sampled every 4 µs, averaged in 128 blocks, and the used current range was 100 pA/V. The substrate and probe positioning, current sampling, and control of electrode potential, were managed by an FPGA card (PCIe-7852R, National Instruments, USA). This was

controlled using a LabVIEW (release 2019, National Instruments) user interface employing the Warwick Electrochemical Scanning Probe Microscopy (WEC-SPM) software.

Supporting Note 4: Details of the spectroscopy/microscopy instrumentation

Scanning electron microscopy (SEM) imaging and energy-dispersive X-ray spectroscopy (EDX) characterization of the WE substrates before and after deposition, as well as of the micropipettes for determining their average diameter, was carried out on SUPRA 55-VP and GeminiSEM 500 systems (Zeiss, Germany). InLens images were obtained at an acceleration voltage of 3 keV to minimize beam damage, with magnifications ranging from roughly 1 k to 40 k dependent on the number of observed depositions and the implemented pipette diameter. Atomic force microscopy (AFM) was performed on a Dimension Icon (Bruker, USA) instrument, using the PeakForce tapping mode with the WE surfaces placed on a motorized stage controlled by AutoMET software. Images were processed and analyzed using the Gwyddion software, and obtained mean values are presented with the corresponding standard deviation unless stated otherwise.

Room temperature Raman spectroscopy was conducted on the WE surfaces after polymer deposition in a confocal set-up using an inVia Raman spectroscope (Renishaw, UK) equipped with a 532 nm laser. It was focused with a 50× objective lens onto the WE surfaces. The data were acquired in a range from 100 nm to 2000 nm using an acquisition time between 10 s and 20 s depending on the size of the observed deposition spot. The laser power was adjusted to maximize the signal to noise ratio, while never exceeding the maximum value of 36.3 mW. The obtained data were processed using OriginPro for background signal removal and noise reduction.

Contact angle measurements were assessed via a KRÜSS Drop Shape Analysis System (DSA100). A glass syringe was loaded with demineralized water or 0.05 M PBS solution and positioned above the electrode surface. Then, a single drop was deposited onto the electrode surface. Each measurement was performed 5 times.

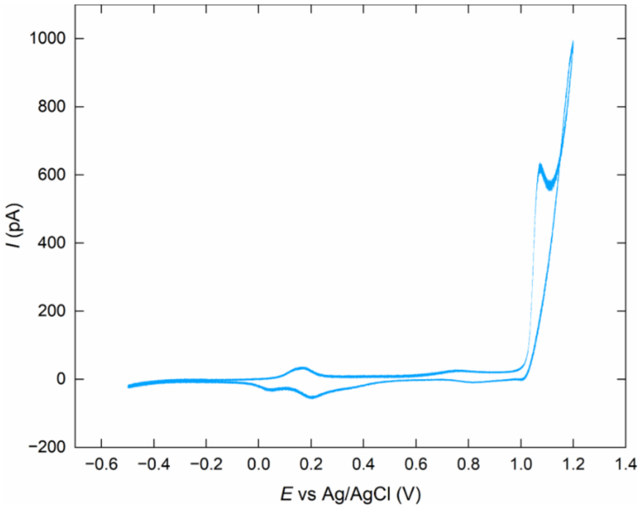

Figure S1: Electrolyte CV data | SECCM cyclic voltammogram (2nd cycle), with a ca. 2 μm diameter tip of a 0.05 M phosphate buffer electrolyte solution on Au/glass at a scan rate of 0.05 V/s, showing the oxidative potential limit prior to oxygen evolution (E > 1.0 V vs. Ag/AgCl$_{QRCE}$).

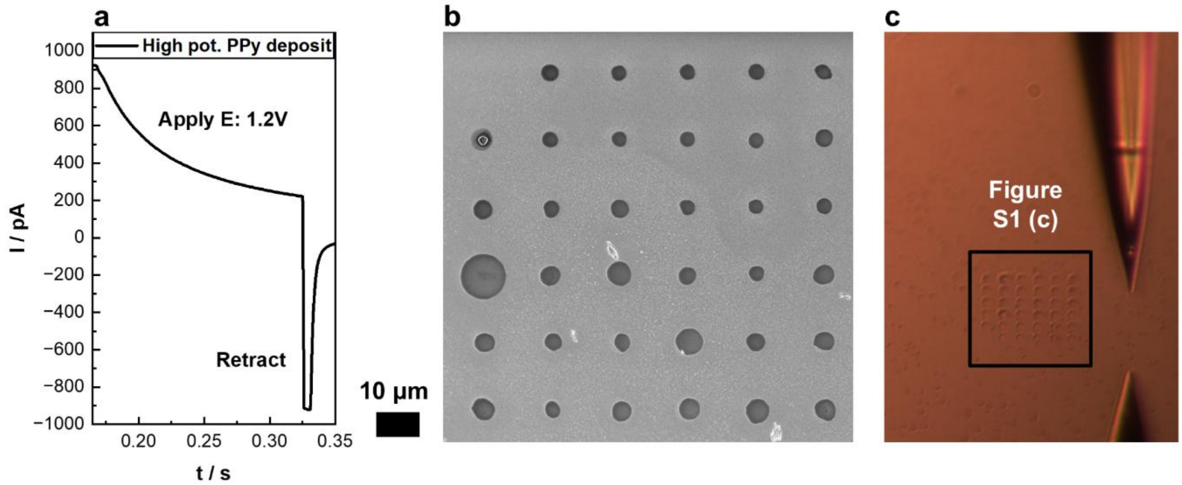

Figure S2: Data for high potential deposits and additional camera images | (a) *i*-*t*-curve of a high potential deposit (E = 1.2 V vs. Ag/AgCl$_{QRCE}$) of PPy using an aqueous phosphate buffer solution electrolyte (c = 0.05 M) on Au/glass with the corresponding 6×6 array SEM micrograph (b). (c) Pixelink camera image of a deposit on the SECCM rig (tip also shown) just after completion of an array fabrication. The displayed measurement used a *ca*. 2 µm diameter tip, an approach rate of 0.5 µm/s, and a retract rate of 3 µm/s.

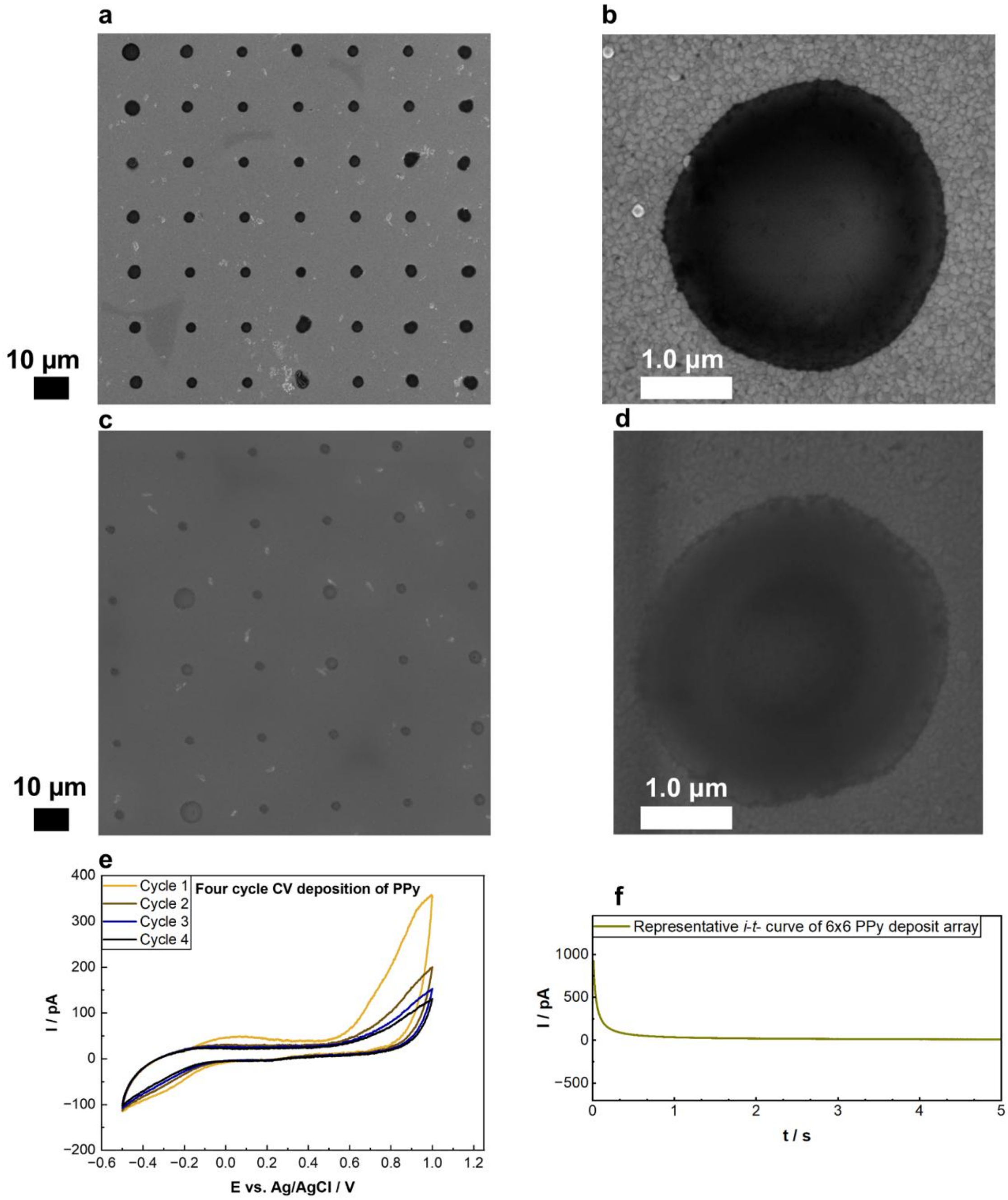

Figure S3: SEM micrographs of fabricated polymer arrays | (a) 7x7 array (15 μm hopping distance) of CV layer-by-layer SECCM deposits of PPy on Au using PB-electrolyte with a representative 1×1 close-up shown in (b). Parameters used for the CV deposits were E_{Approach} = -0.2 V; E_1 = 1.0 V; E_2 = -0.5 V (all referenced vs. Ag/AgCl$_{\text{QRCE}}$); 4 cycles per hop at a scan rate of 1.0 V/s. (c) 6×6 complete array of *i-t* SECCM deposits of PPy on Au using PB-electrolyte without cleaning steps. The first approach in the top left was used for optimizing the approach

distance, and no polymerization potential was applied. A single spot close-up is shown in (d). Parameters used for the *i*-*t*-deposits were $E_{Approach}$ = -0.2 V; E_{it} = 1.0 V (both referenced vs. Ag/AgCl$_{QRCE}$); t_{it} = 200 ms. For each column, t_{it} was increased by 5 s, though no significant increase in deposit diameter was observed. (e) and (f) show the CV- and *i*-*t*- curves of a representative deposit of the respective arrays. All experiments shown used a *ca.* 2 µm diameter tip, an approach rate of 0.5 µm/s, and a retrack rate of 3 µm/s. All micrographs have their respective scale bars present in the bottom left of each image.

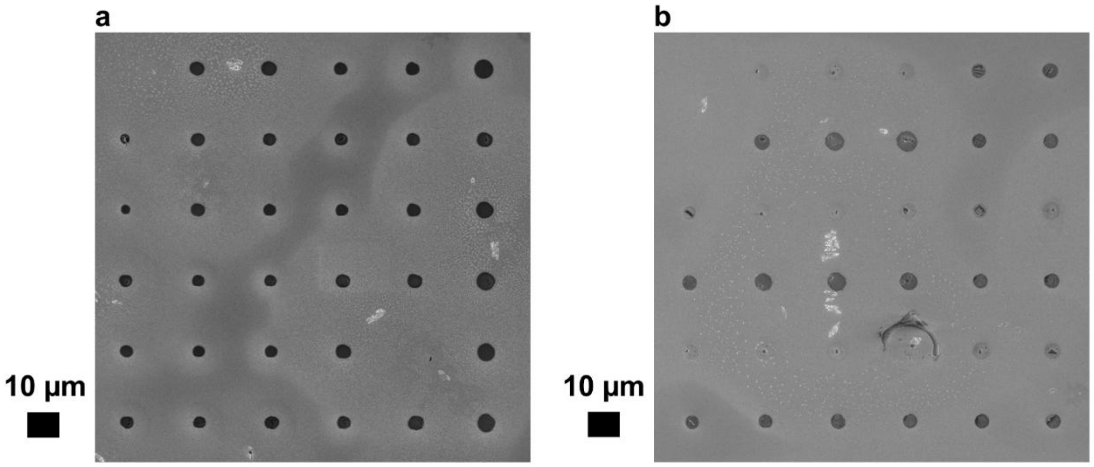

Figure S4: Complete SEM micrographs of PPy arrays using different electrolytes and cleaning steps | (a) 6×6 array SEM micrograph of microscale PB-electrolyte and (b) KNO_3-electrolyte PPy deposits with every second approach being a deposit, the one before and after being a cleaning step, respectively. Parameters for both deposits: $E_{Approach}$ = -0.2 V; E_{it} = 1.0 V; E_{Clean} = 0.2 V (all referenced vs. Ag/AgCl$_{QRCE}$); t_{it} = 1 s; t_{clean} = 1 s. For each row, t_{it} was increased by 30 s, though no significant increase in deposit diameter was observed. Clear contrast differences between deposits and cleaning steps can be observed for the KNO_3-electrolyte sample depicted in (b). The micrographs have their respective scale bars present in the bottom left of each image.

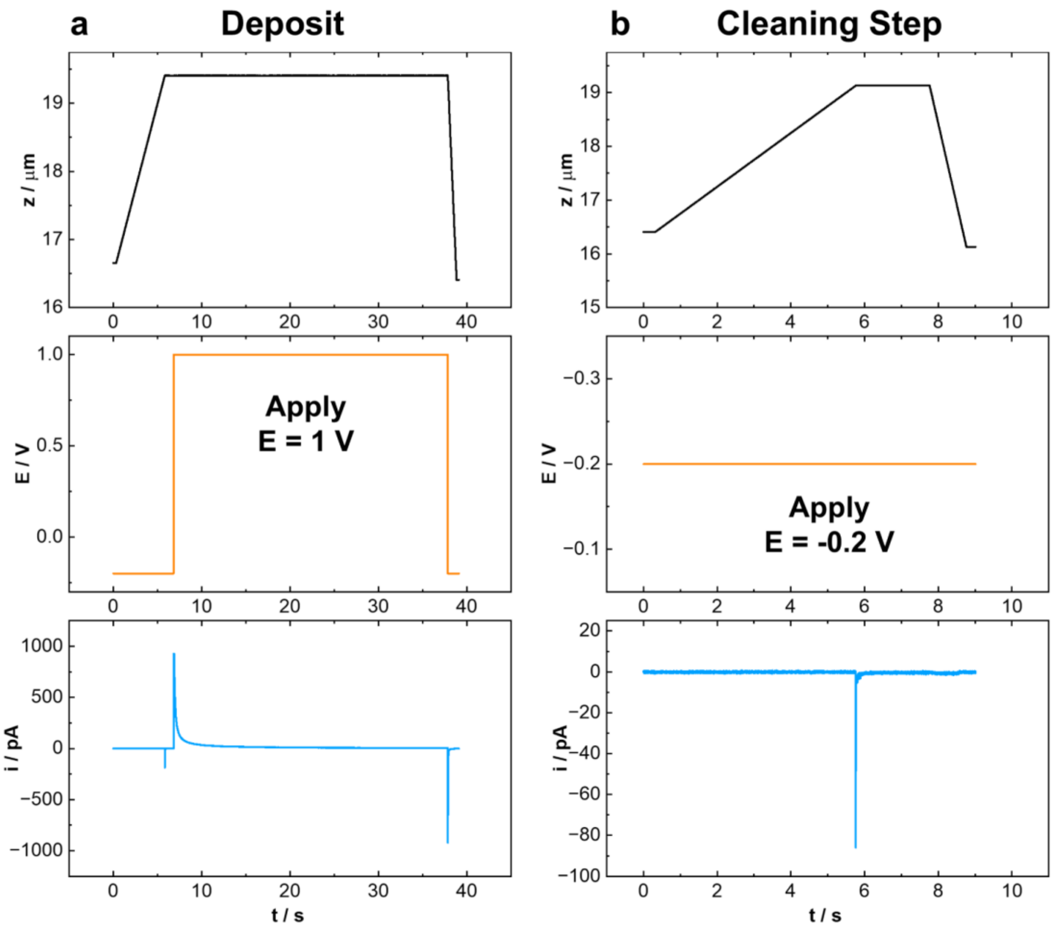

Figure S5: Deposit and cleaning currents alongside z-positions and potential waveforms | From top to bottom: *z*-extension of the pipette, applied deposition potential (1.0 V vs. $Ag/AgCl_{QRCE}$ for (a) and -0.2 V vs. $Ag/AgCl_{QRCE}$ for (b)), and recorded current response over time for (a) a KNO_3-PPy deposit and (b) a KNO_3-PPy cleaning step, each using a *ca*. 2 µm diameter tip.

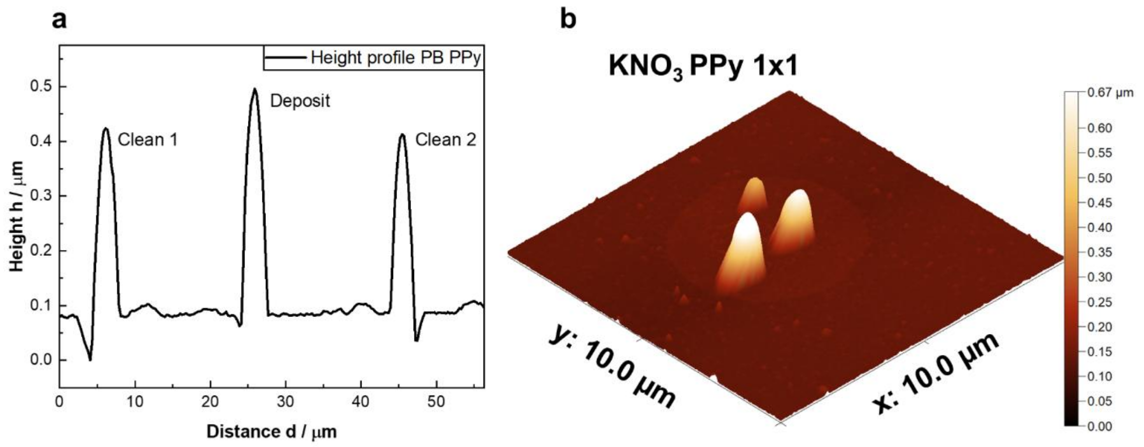

Figure S6: Additional height profile and single spot AFM data | (a) Representative height profile line of a consecutive cleaning step (Clean 1), deposit, and subsequent cleaning step (Clean 2) of a PB-electrolyte PPy *i*-*t*-deposit. (b) AFM data of a representative 1×1 deposit of the discussed KNO_3-electrolyte PPy *i*-*t*-deposit 6×6 array including cleaning steps, as shown in Figure S4. Height profiles extracted and AFM data visualized using the Gwyddion software package.

Supporting Note 5: Further tuning the deposits: Nanoscale pipettes, PANI, DI-rinsing

The parameters of SECCM-microfabrication can be tuned further after fully understanding the impact of PB- and KNO_3-electrolyte on PPy deposits. Reducing the pipette tip diameter resulted in a decreased meniscus size, and thus, a smaller area for polymerisation to occur. Shifting from a ~2 µm diameter to ~200 nm pipette, PPy was deposited in a 6×6 array using a PB-electrolyte, including the implementation of cleaning steps. The electrochemical data of a deposit and cleaning step with the nanoscale pipette having a significantly lower maximum current during the cleaning step ($i_{max,\,clean} < 9$ pA) than the data for cleaning steps using a larger pipette, is shown in SI Figure S6. The resulting polymer array is depicted in SI Figure S7 (a). The EDX data for these deposits confirms the chemistry of the nanoscale deposits is comparable to the microscale samples (SI Figure S8).

In order to determine whether the phosphate intercalation was a property unique to PPy, an additional experiment was performed where aniline (20 mM) was used as the precursor material instead of pyrrole. The aniline was mixed with 0.05 M PB solution and electropolymerized at 0.8 V (vs. Ag/AgCl) to form polyaniline (PANI) in a 6×6 array. This array was also observed using SEM/EDX to see if the oxygen signal intensity was higher than the carbon signal intensity (SI Figure S9). The SEM and EDX data for the PB-electrolyte PANI deposits are comparable to the PB-electrolyte PPy deposits, indicating that the phosphate intercalation is not unique to PPy. It may be of interest to further study this effect on other SECCM polymer microdeposits.

To remove the intercalated phosphate ions from the PPy arrays, the Au/glass sample can be rinsed with DI water. The rinsed and dried deposits can be seen in SI Figure S7 (b-d).

From the SEM micrograph, for the nanoscale deposits, an average deposit diameter of $\bar{d}_{deposit}$ = (0.6 ± 0.2) µm has been fabricated. This is a decrease of 83% compared to the deposits produced using the *ca.* 2 µm diameter pipettes. This confirms that this fabrication tool can be used to produce nanoscale features for applications such as sensor arrays.

Figure S7 (b) shows the SEM image of the deposits in Figure 2 (c) after rinsing with DI water and drying by vacuum. Here, in opposition to Figure 2 (c), the contrast of the cleaning steps clearly differs from the deposition steps for the PB-electrolyte sample. This indicates that there was no polymeric, water-insoluble PPy deposited to the surface in the cleaning steps, and instead water-soluble side products with potential heavy intercalation of phosphate, which were removed. Now, the micrograph looks more similar to the KNO3-electrolyte sample from Figure 2 (d), without KNO_3 crystallization blocking the tip, and leading to crystalline protrusions.

Comparing the Raman spectroscopy data of the PB-electrolyte PPy deposits and cleaning steps after rinsing (SI Figure S10 (a)) to the Raman data before rinsing in Figure 3 (f) confirms that phosphate species have been removed after rinsing. The cleaning steps no longer show any P=O backbone signals. Likewise, the actual deposits now only show the C=C band modes of the conjugated PPy backbone ring structure bands around 1600 cm^{-1} and 1400 cm^{-1}. Figure S10 (b) and (c) display the full 6×6 arrays of the PB-electrolyte and KNO_3-electrolyte PPy deposits after rinsing with DI water.

The 1×1 close-up SEM micrograph data of the PB-electrolyte PPy deposits and cleaning steps after rinsing with DI water depicted in Figure 4 (c) and (d) show detailed correlation with the Raman spectroscopy findings. The cleaning step shows no leftover dark contrasting material, unlike the deposit step which shows high contrast, indicating remaining organic material. Thus, if the desired polymer for deposition is water-insoluble, rinsing with DI water may be useful, especially if the intercalated electrolyte ions are deemed to hinder the final usage of the produced sample. Future work should aim to further observe differences in the electrochemical performance of the polymer deposits based on the used substrate and intercalated electrolyte ions.

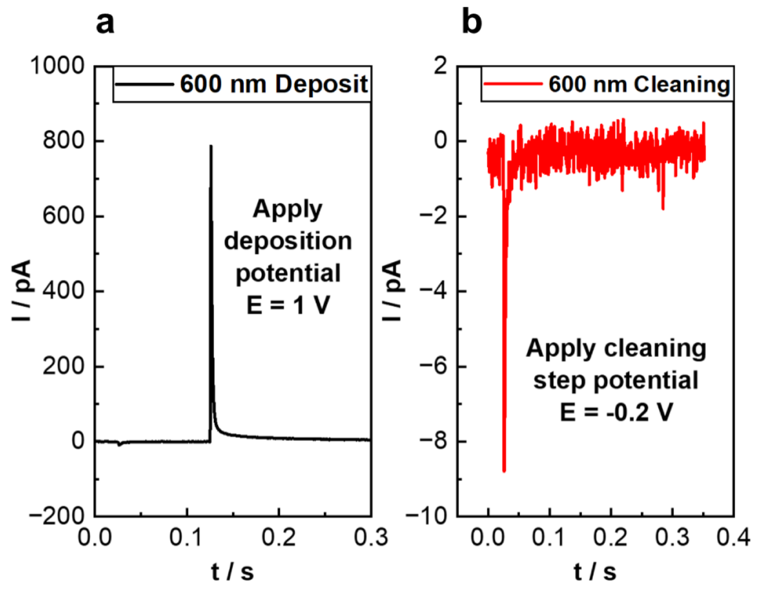

Figure S7: SECCM *i-t*-curve of nanoscale PPy deposit and cleaning step | (a) depicts a representative approach (E = -0.2 V vs. Ag/AgCl$_{QRCE}$), dwelling for 100 ms, and 200 ms deposit (E = 1.0 V vs. Ag/AgCl$_{QRCE}$) before retraction of a nanoscale PPy deposition using an aqueous phosphate buffer solution electrolyte (c = 0.05 M) on Au/glass. (b) shows the subsequent cleaning step at a holding potential of E = -0.2 V vs. Ag/AgCl$_{QRCE}$ for 200 ms, after which retraction of the pipette probe occured.

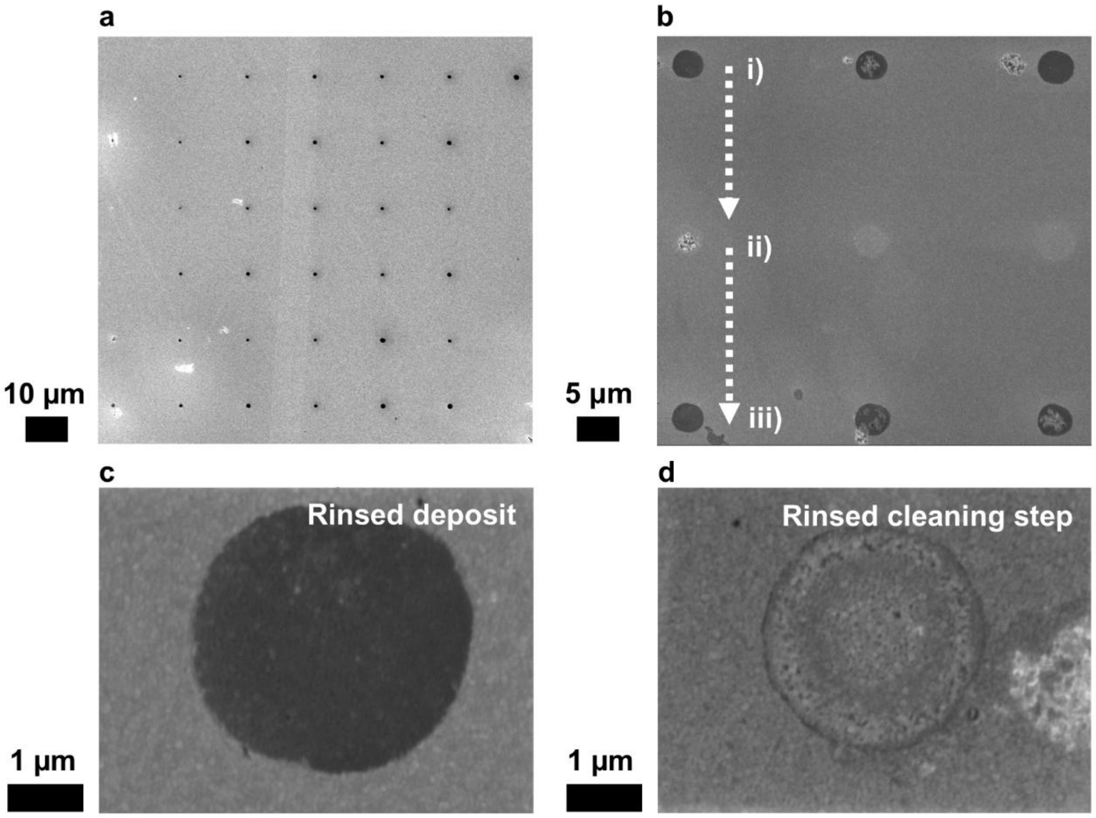

Figure S8: Nanoscale and DI-rinsed PPy | (a) SEM micrographs of a 6 x 6 PPy deposit array employing a nanoscale pipette (d = 200 nm). (b) PB electrolyte deposits i) and iii) and cleaning steps ii) after rinsing with DI water and vacuum-drying the substrate. (c) SEM micrograph of a single representative PPy deposit after rinsing and (d) shows the same for a representative cleaning step after subsequent rinsing.

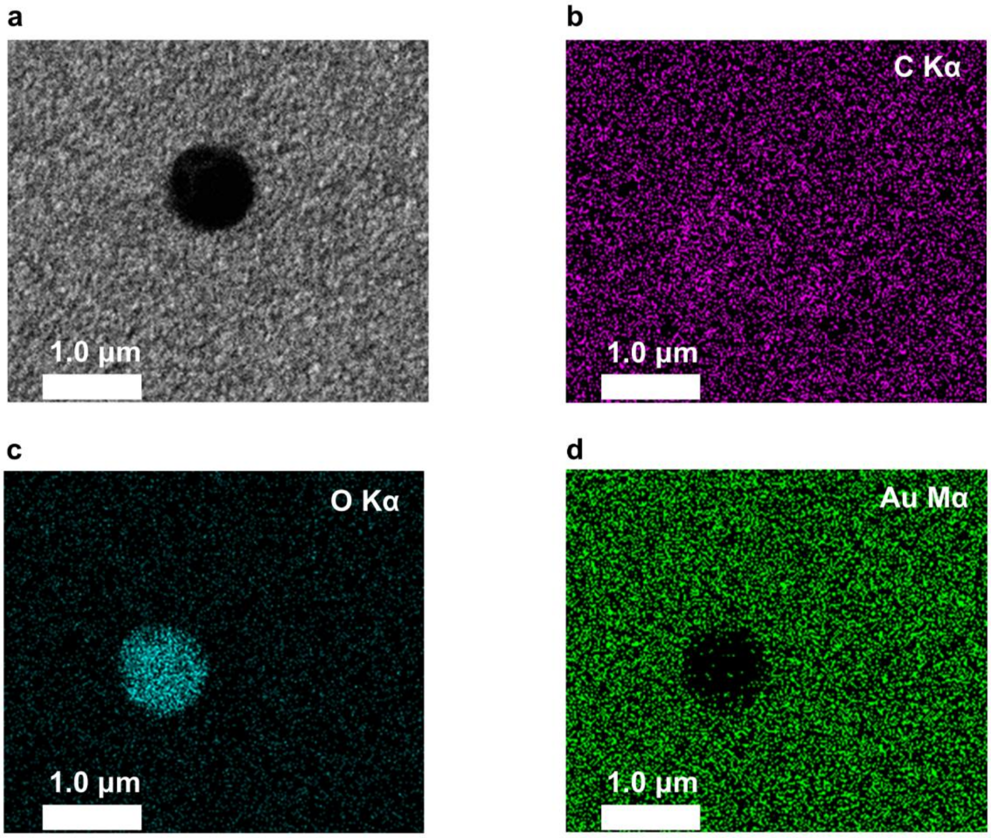

Figure S9: EDX characterization of nanoscale PPy | Depicted are (a) the SEM micrograph and the corresponding EDX maps (C Kα (b), O Kα (c), Au Mα (d)). Scale bars are located in the bottom left of each image. Due to the small size of the deposit and the required beam exposure time for obtaining a significant amount of counts, a small drift between the SEM micrograph and EDX images is present.

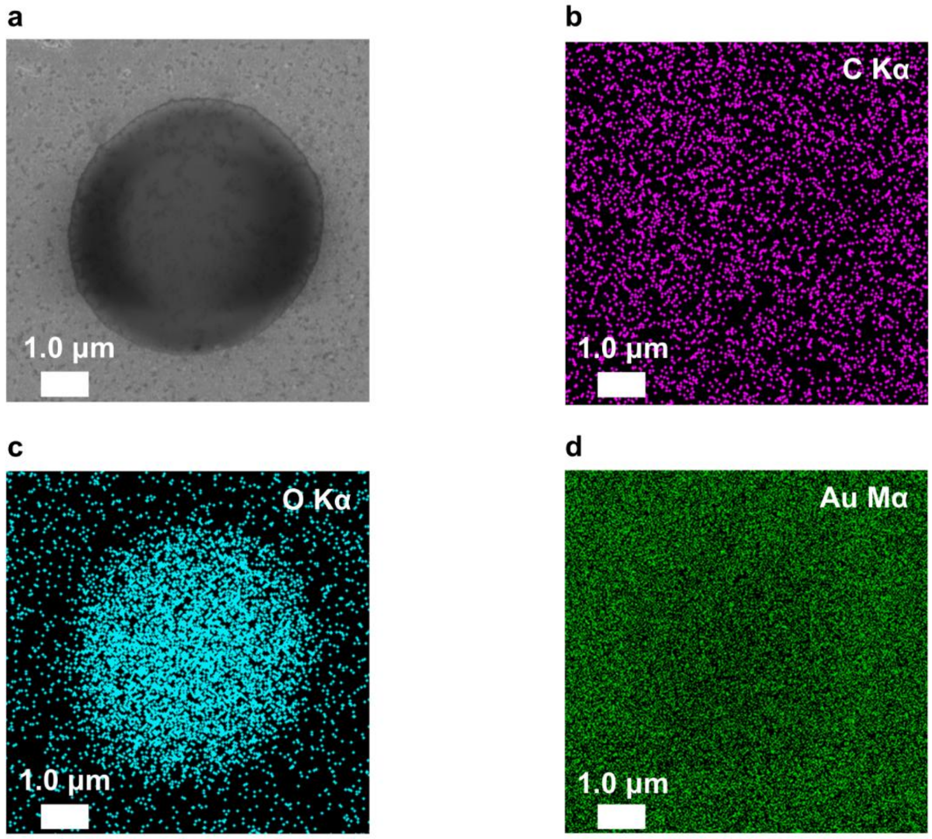

Figure S10: EDX characterization of PANI deposit | Depicted are (a) the SEM micrograph and the corresponding EDX maps (C Kα (b), O Kα (c), Au Mα (d)) of a PB-electrolyte PANI SECCM *i*-*t*-deposit. Scale bars are located in the bottom left of each image.

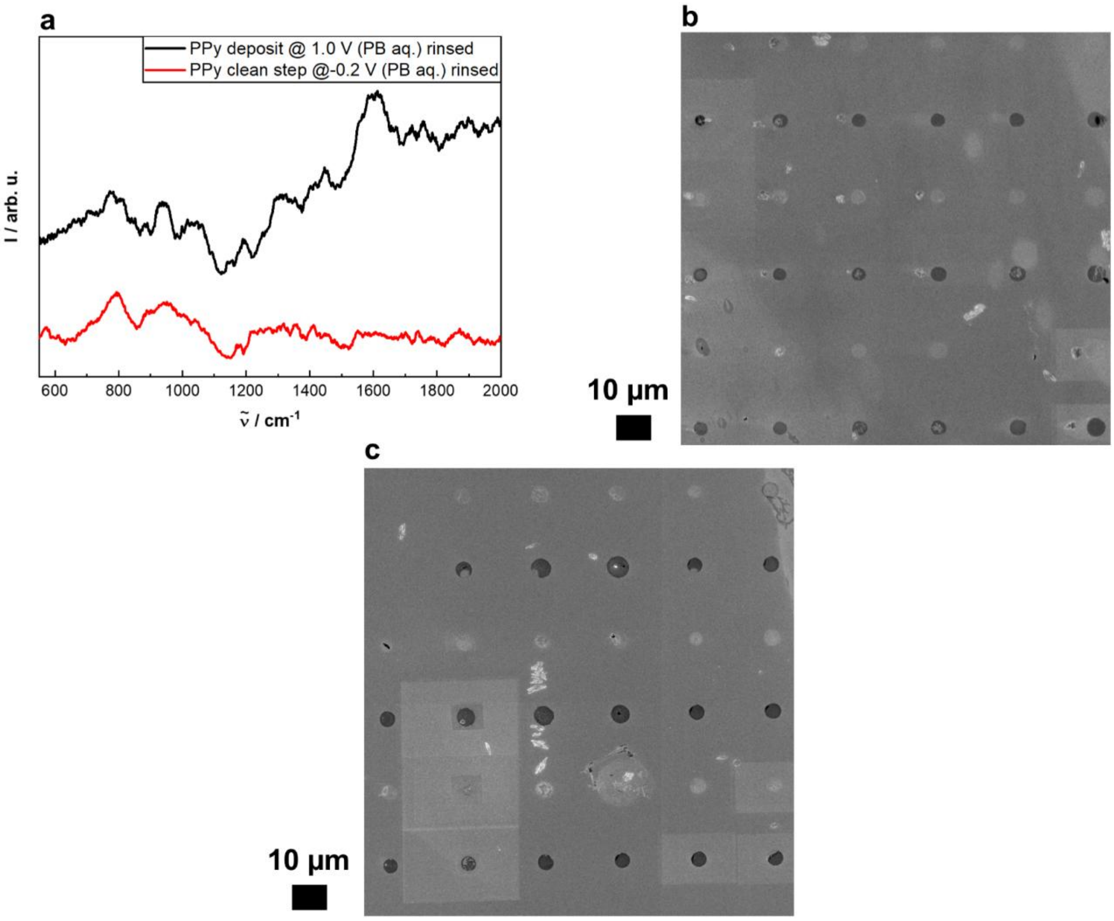

Figure S11: Additional characterization of rinsed PB-electrolyte PPy deposits | (a) Raman spectroscopy data of a single representative PB-electrolyte PPy deposit (black) and cleaning step (red). Data manipulation (background removal, smoothing) was performed using the OriginPro software package. (b) depicts the complete 6×6 SEM micrograph of the sample that was analyzed using Raman spectroscopy, and (c) shows the complete 6×6 SEM micrograph of a reference sample using KNO_3-electrolyte after rinsing with DI water. Areas of low contrast are present due to previous 1×1 single spot investigations of the same sample before rinsing with DI water.

Supporting Note 6: Details regarding the additional characterization of B:CNW

The results of the contact angle measurements can be seen in SI Figure S11. Ti/Au exhibits contact angles of 30.8° ± 1.1° and 57.4° ± 0.3° in DI and 0.05 M PB. In contrast, B:CNW shows higher hydrophobic potential than Ti/Au in both DI and 0.05 M PB, resulting in contact angles of 134.6° ± 1.6° and 125.7° ± 0.6° respectively (n = 10). (a) depicts the SEM close-up micrograph of the B:CNW structure, confirming the lamellar, disordered assembly of carbon nanowalls. Surprisingly, comparing the contact angles of H_2O, 0.05 M PB, and 0.05 M KNO_3 on B:CNW (b) with those on Au/glass (c), the B:CNW sample shows significantly higher contact angles for every used analyte, with the difference for PB-electrolyte being 67.6°. The effect of high surface charge on the B:CNW clearly outweighs the porosity induced by the lamellar surface structure of the system, making B:CNW an excellent candidate for precise meniscus-confined polymer fabrication using SECCM. Even without the presence of any salt, the wetting of pure H_2O on B:CNW is minimal and may allow for polymerization experiments merely using the precursor dissolved in water. Droplets can slowly dissolve in the grooves of the B:CNW structure over long stretches of time, so deposition processes should be performed rapidly for the fabrication on B:CNW.

For CV-tests, PB electrolyte was chosen over KNO_3 for the tests, as the previous experiments have shown the irregular growth of crystals to be a common occurrence around the polymerization potential of PPy, which could cause even more polymer growth issues considering the rough nature of the B:CNW substrate. The CV results performed with a *ca*. 2 micron pipette probe are shown in Figure S12, displaying the shift of the OER occurring at potentials over 1.2 V instead of 1.0 V, as observed for Au/glass, where Au catalyzes the reaction. Thus, it was deemed feasible to perform CV-electropolymerization tests from -0.5 V to 1.2 V at the usual 1.0 V/s scan rate, to allow for more time spent in the potential range where

oxidative polymerization can occur. No additional CV peaks were observed compared to the measurements performed on Au/glass (SI Fig. S2).

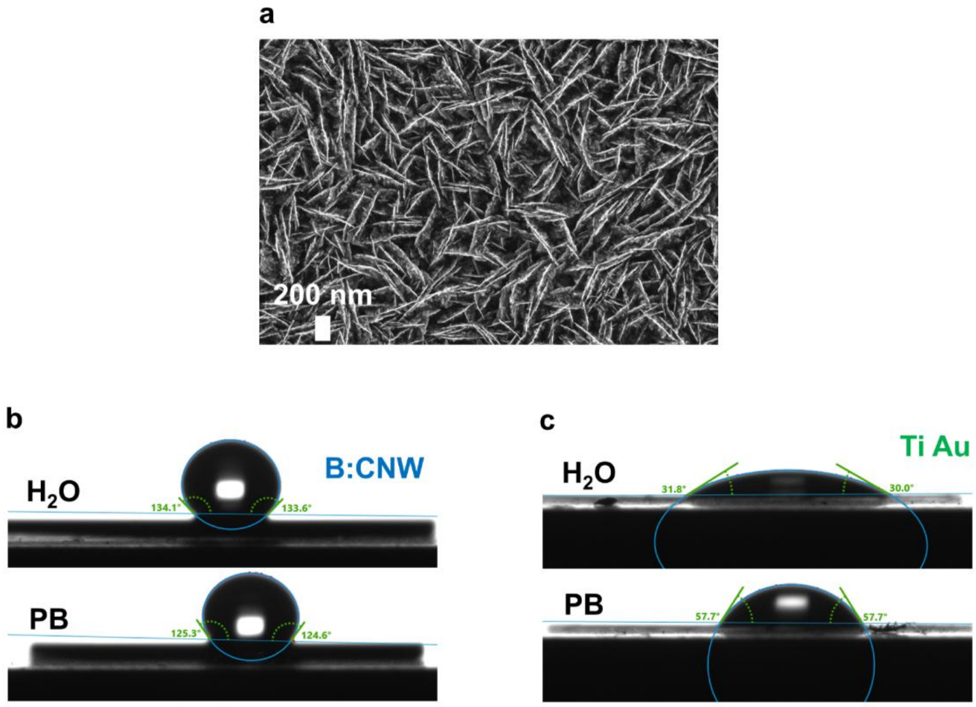

Figure S12: SEM and contact angle tests of B:CNW (and Au) substrates | (a) High-magnification SEM micrograph of a B:CNW surface deposited onto a p-type (100) silicon substrate. (b) and (c) show contact angle measurements of B:CNW and the Au surface substrate types respectively, with two different solvents/electrolytes being analyzed (from top to bottom: H_2O and 0.05 M PB (aq.). Depicted are representative results of the experiments.

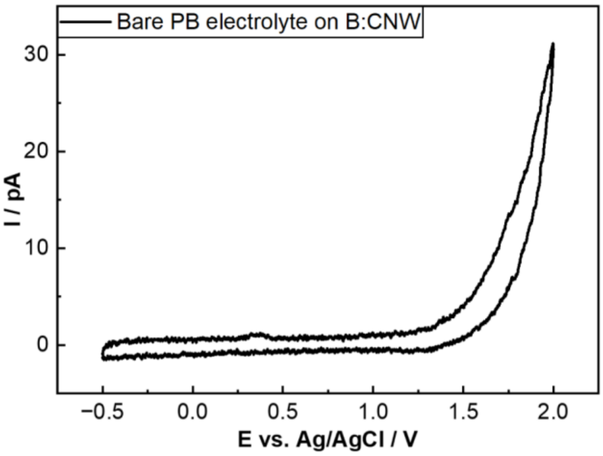

Figure S13: CV potential window checking of B:CNW | Depicted is a representative CV curve of bare 0.05 M PB-electrolyte without precursor solution on the B:CNW substrate recorded at a scan rate of 1.0 V/s, used for determining the potential window for CV-electropolymerization. Used potentials were: E_{start} = -0.2 V; E_1 = 2.0 V; E_2 = -0.5 V (all vs. Ag/AgCl$_{QRCE}$).

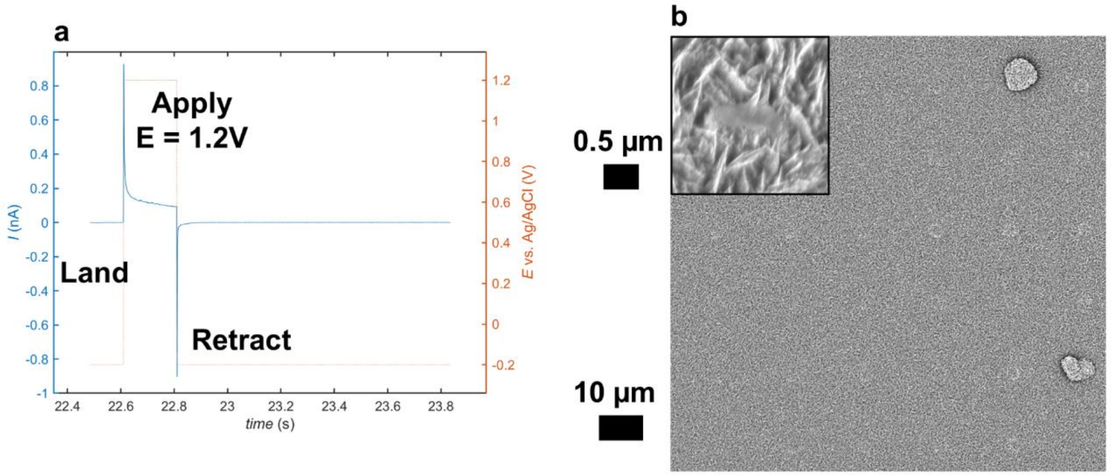

Figure S14: Electrochemical and SEM data of *i-t* deposits of PPy on B:CNW | (a) *i*-*t*-curve of a representative approach (E = -0.2 V vs. Ag/AgCl$_{QRCE}$) and deposit (E = 1.2 V vs. Ag/AgCl$_{QRCE}$) of a PPy deposition using an aqueous phosphate buffer solution electrolyte (c = 0.05 M) on B:CNW. The deposition potential was determined via CV potential window

checking. (b) depicts the SEM micrograph of the fabricated 6×6 PPy array with a high-magnification inset of a single, faintly visible deposit.

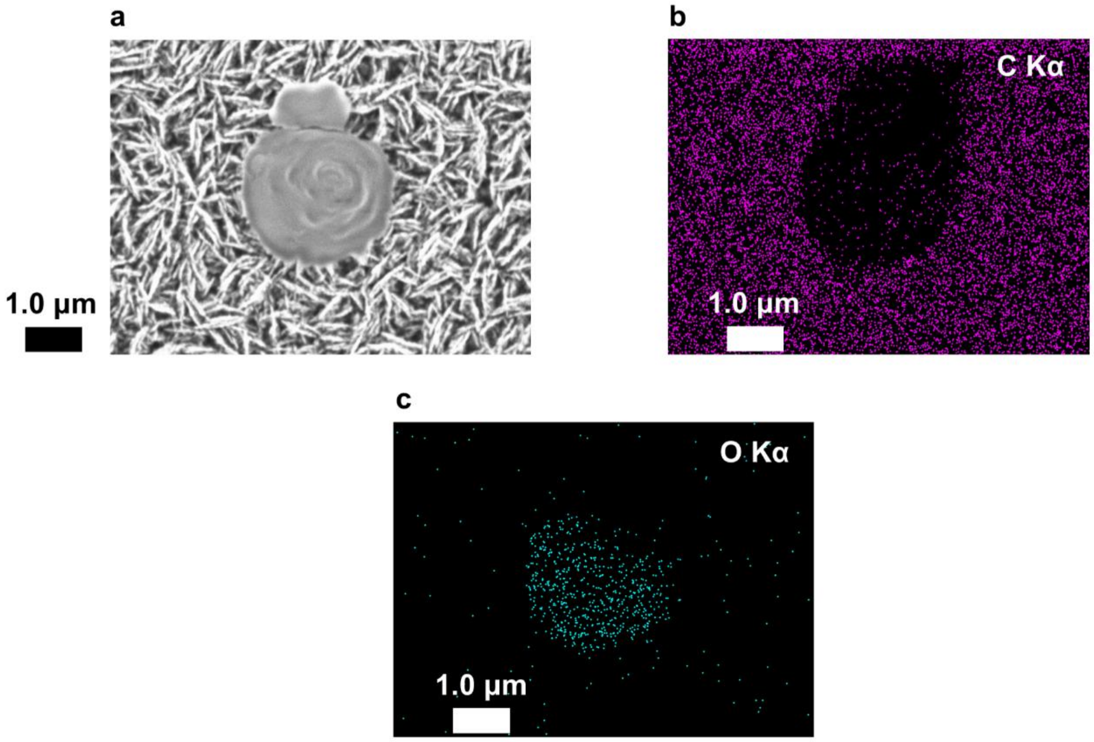

Figure S15: EDX single-spot analysis of PB-electrolyte PPy deposit on B:CNW | Depicted are (a) the SEM micrograph and the corresponding EDX maps (C Kα (b), O Kα (c)). Scale bars are located in the bottom left of each image. Due to low signal to noise-ratio caused by the background signal of the carbon-rich B:CNW substrate, the visual clarity of the EDX figures was improved using the variance filter in the ImageJ software, employing a radius of 1.5 pixels.
